# Heme oxygenase is not involved in the anti-proliferative effects of statins on pancreatic cancer cells

**DOI:** 10.1186/s12885-016-2343-9

**Published:** 2016-05-12

**Authors:** K. Vanova, S. Boukalova, H. Gbelcova, L. Muchova, J. Neuzil, R. Gurlich, T. Ruml, L. Vitek

**Affiliations:** Institute of Medical Biochemistry and Laboratory Diagnostics, 1st Faculty of Medicine, Charles University in Prague, Katerinska 32, Prague 2, 120 00 Czech Republic; Institute of Biotechnology, Czech Academy of Sciences, Videnska 1083, Prague 4, 142 20 Czech Republic; Department of Biochemistry and Microbiology, University of Chemistry and Technology, Technicka 1905/5, Prague 6, 160 00 Czech Republic; School of Medical Science, Griffith University, Parklands Avenue, 4222 Southport, QLD Australia; Department of Surgery, University Hospital Kralovske Vinohrady and Charles University in Prague, Srobarova 50, Prague 10, 100 34 Czech Republic; 4th Department of Internal Medicine, 1st Faculty of Medicine, Charles University in Prague, Katerinska 32, Prague 2, 120 00 Czech Republic

**Keywords:** Heme, Heme oxygenase, Pancreatic cancer, Statins

## Abstract

**Background:**

Pancreatic cancer is recognized as one of the most fatal tumors due to its aggressiveness and resistance to therapy. Statins were previously shown to inhibit the proliferation of cancer cells *via* various signaling pathways. In healthy tissues, statins activate the heme oxygenase pathway, nevertheless the role of heme oxygenase in pancreatic cancer is still controversial. The aim of this study was to evaluate, whether anti-proliferative effects of statins in pancreatic cancer cells are mediated *via* the heme oxygenase pathway.

**Methods:**

In vitro effects of various statins and hemin, a heme oxygenase inducer, on cell proliferation were evaluated in PA-TU-8902, MiaPaCa-2 and BxPC-3 human pancreatic cancer cell lines. The effect of statins on heme oxygenase activity was assessed and heme oxygenase-silenced cells were used for pancreatic cancer cell proliferation studies. Cell death rate and reactive oxygen species production were measured in PA-TU-8902 cells, followed by evaluation of the effect of cerivastatin on GFP-K-Ras trafficking and expression of markers of invasiveness, osteopontin (SPP1) and SOX2.

**Results:**

While simvastatin and cerivastatin displayed major anti-proliferative properties in all cell lines tested, pravastatin did not affect the cell growth at all. Strong anti-proliferative effect was observed also for hemin. Co-treatment of cerivastatin and hemin increased anti-proliferative potential of these agents, *via* increased production of reactive oxygen species and cell death compared to individual treatment. Heme oxygenase silencing did not prevent pancreatic cancer cells from the tumor-suppressive effect of cerivastatin or hemin. Cerivastatin, but not pravastatin, protected Ras protein from trafficking to the cell membrane and significantly reduced expressions of *SPP1* (*p* < 0.05) and *SOX2* (*p* < 0.01).

**Conclusions:**

Anti-proliferative effects of statins and hemin on human pancreatic cancer cell lines do not seem to be related to the heme oxygenase pathway. While hemin triggers reactive oxygen species-induced cell death, cerivastatin targets Ras protein trafficking and affects markers of invasiveness.

## Background

Pancreatic cancer has a very poor prognosis mainly due to late diagnosis of already advanced tumors, often with metastases to distant organs. Since high resistance to therapy aggravates the treatment outcomes, new efficient treatment modalities and therapy targets are under investigation.

Statins, competitive inhibitors of 3-hydroxyl-methylglutaryl coenzyme A (HMG CoA) reductase, are widely used for treatment of hypercholesterolemia. However, their therapeutic role surpasses the cholesterol lowering capacity, utilizing anti-inflammatory, anti-oxidant and anti-thrombotic actions [1]. Additionally, several studies suggested the anti-proliferative role of statins in various cancer cell lines, including lung [2], colorectal [3] and pancreatic cancer [4–7]. These effects could be partly mediated by the depletion of several important intermediates of cholesterol biosynthesis involved in posttranslational protein prenylation. This process is especially important for modification of small GTPases, such as Ras [8, 9], which is essential for their translocation from cytoplasm to the cell membrane, affecting thus their cell proliferating activities [1] *via* targeting several important signal transduction pathways [10–12]. The association of activation mutations in the *K*-*ras* oncogene with pancreatic cancer is well established, being found in more than 90 % of human pancreatic cancers [13]. We previously reported that most statins protect green fluorescent protein (GFP)-K-Ras from its anchoring to the cell membrane, affecting the signaling pathways and leading to suppression of cancer cell growth in pancreatic cancer cells in vitro [4].

Heme oxygenase (HMOX), the key enzyme in heme metabolism, catalyzes the degradation of heme to equimolar quantities of CO, free iron and biliverdin, which is subsequently converted to bilirubin [14]. While the induction of HMOX1 represents a key biological process in adaptive response to cellular stress and displays anti-inflammatory, anti-apoptotic and anti-oxidative actions [14–17], its role in cell proliferation and tumor progression is still controversial [18, 19]. Some studies suggested that statins can upregulate the *HMOX* gene expression in a cell- and species-specific manner [20–24], and they exert some of their protective effects *via* this pathway [21]. However, the upregulation of HMOX1 in pancreatic cancer cells was previously connected to worsened treatment outcome [25].

The aim of this study was to evaluate anti-proliferative effects of statins with respect to their possible role in modulation of HMOX pathway in pancreatic cancer in vitro. Hemin, a strong HMOX1 inducer [26], was used a control compound. Further, we investigated the effects of cerivastatin on targeting the GFP-K-Ras protein trafficking, as well as the regulation of invasiveness of pancreatic adenocarcinoma cells in vitro, elucidating the potential involvement of statins in pancreatic cancer therapy.

## Methods

### Chemicals

Cerivastatin, pravastatin and fluvastatin were purchased from LKT Laboratories, Inc (USA), lovastatin and simvastatin from Santa Cruz Biotechnology (Dallas, TX, USA). Bovine serum albumin (BSA), hemin, reduced nicotinamide adenine dinucleotide (NADPH), sulfosalicylic acid, Dulbecco’s Modified Essential Media (DMEM), and RPMI-1640 were purchased from Sigma-Aldrich (St. Louis, MO, USA). Fetal bovine serum (FBS) and L-glutamine (L-Glu) were purchased from Biosera (Boussens, France), 15-deoxy-Δ-12,14-prostaglandin J2 (PGJ2) was purchased from Merck (Darmstadt, Germany).

### Cell culture

For cell culture studies, the following pancreatic cancer cell lines were used: PA-TU-8902 (DSMZ, Braunschweig, Germany), MiaPaCa-2 and BxPC-3 (ATCC, Manassas, VA, USA). All cell lines were maintained and grown in a humidified atmosphere containing 5 % CO_2_ at 37 °C. PA-TU-8902 and MiaPaCa-2 were cultured in DMEM supplemented with 10 % FBS, antibiotics and 1 % L-Glu, BxPC-3 in RPMI-1640 supplemented with 10 % FBS, antibiotics and 2 % L-Glu. For all experiments, medium with reduced content of FBS to the final concentration of 0.5 % was used. All statins in the study were used at 12 μM (corresponding to IC_50_ of simvastatin for MiaPaCa-2 cells after 24 h incubation [4]) diluted in methanol (vehicle) and hemin (methemalbumin) was prepared as previously described and used in the final concentration of 30 μM (pH = 7.4) [26].

Ethical approval for work on cell lines was not required by our Institution.

### HMOX RNA interference (RNAi)

Pancreatic cancer cells were transfected with 10 pmol of HMOX1 esiRNA and 10 pmol of HMOX2 esiRNA (Sigma-Aldrich) per 5 x 10^3^ seeded cells using the Lipofectamine RNAiMAX reagent (Life Technologies, Carlsbad, CA, USA) for 24 h in ATB-free DMEM medium. The esiRNA Universal control was used as negative control in all experiments. Data were expressed as % of esiRNA Universal control (Sigma-Aldrich).

### Cell proliferation assay

For the cell proliferation assay, cells were seeded into 96 well (5–12.5 x 10^4^ cells per ml according to the cell line) and kept at 37 °C and 5 % CO_2_. After 24 h, cells were treated with statins or/and hemin, followed by the MTT test (Sigma-Aldrich) as a general cell proliferation assay. As we experienced difficulties with hemin-treated samples using MTT test due to interfering effects of hemin, we further used the more sensitive CellTiter-Glo Luminescent Cell Viability Assay (Promega, Fichburg, WI, USA). Both tests were used according to the manufacturer's instructions. Results were expressed as % of controls.

### HMOX activity measurement

Cells in plates were treated with statins and hemin. After 12 h, cells were washed twice with ice-cold phosphate buffer and finally collected into freshly added phosphate buffer and centrifuged. The pellet was resuspended in 150 μl of 0.1 M potassium phosphate buffer (pH = 7.4) and sonicated with an ultrasonic cell disruptor (Model XL2000, Misonics, Farmingdale, NY, USA). The protein concentration was assessed using the DC™ Protein Assay (Bio-Rad Laboratories, Hercules, CA, USA) according to the manufacturer's instruction. A total of 0.15 mg of protein was incubated for 15 min at 37 °C in CO-free septum-sealed vials containing 20 μl of 4.5 mM NADPH as previously described [27]. The amount of CO generated by HMOX activity was quantified by gas chromatography with a reduction gas analyzer (Peak Laboratories LLC, Mountain View, CA, USA) and calculated as pmol CO/h/mg protein. Five μM PGJ2 was used as a positive control of heme regulation. Results were expressed as % of control.

### Western blot analyses

For protein expression analyses, cells were transfected with esiRNA universal control or esiRNA HMOX1/2 as mentioned previously. After 24 h, cells were treated with 30 μm hemin for 20 h. Hemin treatment was used to upregulate HMOX1 protein expression to cumulate detectable levels of HMOX1 protein. Thirty μg of total protein were separated on 12 % polyacrylamide gel and then transferred to nitrocellulose membrane (Bio-Rad Laboratories). After blocking in Tween-PBS with 5 % milk (Sigma-Aldrich) for at least 1 h, membranes were incubated with HMOX1 antibody (1:1000; Thermo Fisher, Rockford, IL, USA), or β-actin (1:1000; Cell Signaling Technology, Danvers, MA, USA) overnight at 4 °C. After washing, membranes were incubated with anti-mouse IgG-HRP (Abcam, Cambridge, UK) for 1 h. Immunocomplexes on the membranes were visualized with ECL Western Blotting Detection Reagents (Cell Signaling Technology).

### Real-time PCR analysis of mRNA

#### HMOX1 expression

Cells grown in plates were treated with statins, hemin or PGJ2. After 4 h, they were washed twice with ice-cold PBS and collected in the lysis buffer. Total cell RNA was isolated using Perfect Pure RNA Cultured Cell Kit (5Prime, Gaithersburg, MD, USA) and cDNA was generated using High Capacity RNA-to-cDNA Master Mix (Life Technologies) according to the manufacturer’s instructions. Real-time PCR for *HMOX1* (OMIM *141250) and *HMOX2* (OMIM *141251) was performed using the SYBR master mix (Life Technologies) according to the manufacturer’s instructions with optimized primers (Generi Biotech, Hradec Králové, Czech Republic). Results were calculated using the comparative Ct method with *HPRT* as a house-keeping gene and were expressed as % of control.

### Markers of invasiveness

Cells were treated for 12, 24 and 48 h with individual statins. Total RNA was collected and cDNA generated as mentioned above. For real-time qPCR, cDNA corresponding to 10 ng of starting total RNA was diluted with water in 3.6 μl; 0.2 μl of the combined 10 μM forward and reverse primers were added and, finally, 3.8 μl of 2x iTaq Universal SYBR Green Supermix (Bio-Rad Laboratories) was added. The reaction was carried out using the Eco real-time PCR system (Illumina, San Diego, CA, USA) using three-step PCR. The relative mRNA expression levels of osteopontin (secreted phosphoprotein 1, *SPP1*, OMIM*166490) and sex-determining region Y-related HMG box 2 (*SOX2*, OMIM*184429) were calculated using the comparative Ct (ΔΔCt) method, with ribosomal phosphoprotein (P0, OMIM*180510) as a reference gene.

Sequences of primers used for real-time PCR: *SPP1* forward, AGA CCT GAC ATC CAG TAC CCT, reverse - CAA CGG GGA TGG CCT TGT AT; *SOX2* forward - AGG ACC AGC TGG GCT ACC CG, reverse - GCC AAG AGC CAT GCC AGG GG.

### Apoptosis evaluation

Apoptosis was quantified using the annexin V-FITC method, which detects phosphatidyl serine externalized in the early phases of apoptosis, in combination with propidium iodide (PI) staining. After exposure to cerivastatin and/or hemin, floating and attached cells were collected, washed with PBS, re-suspended in 100 μl binding buffer and incubated for 20 min at room temperature with 0.3 μl annexin V-FITC (Apronex, Vestec, Czech Republic). PI (10 μg/ml) was added directly before flow cytometry analysis (BD FASC Calibur, BD, Franklin Lakes, NJ, USA). Annexin V positive (An+) and PI negative (PI-) are cells in early apoptosis, An + and/or PI positive (PI+) are cells in late apoptosis or post-apoptotic necrosis.

### Reactive oxygen species (ROS) generation

For assessment of ROS generation, dichlordihydrofluorescein diacetate (H2DCFDA) (Life Technologies) was used. After treatment, cells were washed and exposed to 10 μM H2DCFDA in 37 °C for 20 min. Cells were then washed, lysed and the fluorescent signal at 492/520 (Ex/Em) was evaluated in 100 μl aliquots. Total fluorescence was related to protein concentration. Results were expressed as % of control.

### Ras protein translocation assay

PA-TU-8902 cells were seeded in dishes with glass bottom 6 h before transfection by pEGFP-KrasWT (GFP – green fluorescent protein, WT-wild type) plasmid prepared as described previously [4]. Transfection was carried out using FuGene HD according to the manufacturer’s instructions. Cerivastatin (12 μM), pravastatin (12 μM) and hemin (30 μM) were added 12 h post transfection and the cells incubated with the agents for 24 h. Intracellular localization of the GFP-K-Ras protein was visualized by confocal microscopy, using a spinning disk confocal microscope (Olympus, Tokyo, Japan; Andor, Belfast, UK) equipped with solid state laser (488 nm for continual excitation). Emission was collected through a single-band filter (BrightLine® FF01-525 nm, Semrock Inc., NY, USA). The images were obtained and analyzed with the iQ2 software (Andor).

### Statistical analysis

All data were expressed as mean ± SD. For normally distributed datasets, one-way ANOVA with post-hoc Holm-Sidak test for multiple comparisons was used for analysis. For non-normally distributed data and small datasets (n ≤ 6), Mann–Whitney rank sum test and Kruskal-Wallis ANOVA with Dunn’s test for multiple comparisons were used. *P*-values less than 0.05 were considered statistically significant.

All datasets of the results discussed in the manuscript are available on request.

## Results

### Statins exert different anti-proliferative on pancreatic cancer cell lines

**Fig. 1:**
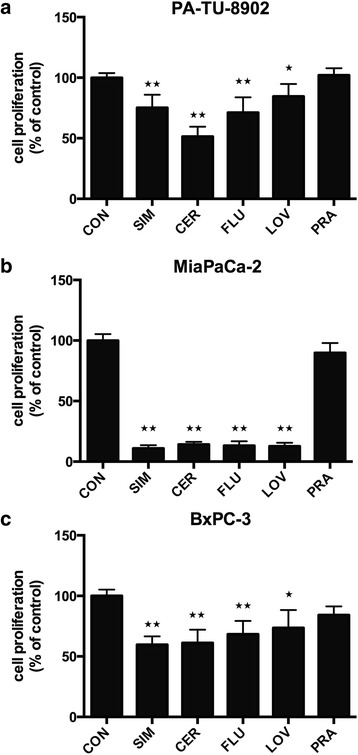
Anti-proliferative effect of statins on pancreatic cancer cells. The effect of simvastatin, cerivastatin, fluvastatin, lovastatin and pravastatin on cell proliferation was measured using MTT test in **a**) PA-TU-8902, **b**) MiaPaCa-2, and **c**) BxPC-3 after 48 h of treatment. Statin concentration = 12 μM *p < 0.01, **p < 0.0001 vs. control cells. CON, control cells; SIM, simvastatin; CER, cerivastatin; FLU, fluvastatin; LOV, lovastatin; PRA, pravastatin

The inhibitory effects of individual statins on proliferation were assessed using cultured human pancreatic cancer cells. Except pravastatin, all selected statins, used at 12 μM, showed significant anti-proliferative effects on growth of tested cancer cells after 48 h of treatment (Fig. 1). Various statins exhibited different anti-proliferative potential covering the spectrum from the most effective cerivastatin and simvastatin, followed by fluvastatin and lovastatin, to ineffective pravastatin. Additionally, we observed significantly different sensitivity for particular pancreatic cancer cell lines. The most sensitive cell line was MiaPaCa-2, bearing activating K-*ras* mutation in codon 12 (34G > T) [28]. By contrast, the other cell line PA-TU-8902 with another activating mutations in K-*ras* oncogene in codon 12 (35G > T) [29] revealed to be more resistant to statin treatment. BxPC-3 cells, featuring wild-type *K-ras* oncogene and overexpressing cyclo-oxygenase-2 [28], were slightly more sensitive than PA-TU-8902 cells to several statins and significantly more resistant to statin treatment than MiaPaCa-2 cells (Fig. 1). The most efficient statin was cerivastatin, which decreased cell proliferation as compared to untreated controls to 51 ± 8 %, 61 ± 10 %, and 14 ± 2 % following 48-h treatment of PA-TU-8902, BxPC-3 and MiaPaCa-2 cells, respectively, with *p* < 0.0001 for all comparisons.

### Statins do not affect HMOX expression and activity in human pancreatic cancer cells

**Fig. 2:**
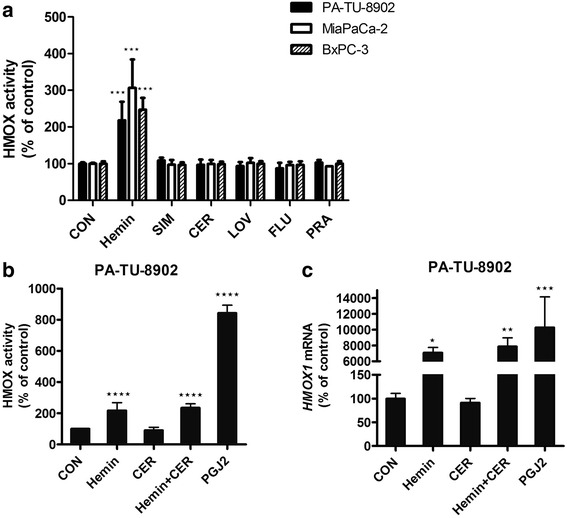
The effect of statins on activity and expression of HMOX in pancreatic cancer cells **a**) HMOX activity was measured in PA-TU-8902, MiaPaCa-2 and BxPC-3 pancreatic cancer cell lines after 12 h of statin treatment (12 μM). **b** HMOX activity after 12 h of treatment were measured in PA-TU-8902, and **c**) *HMOX1* mRNA after 4 h of treatment. Hemin (30 μM) and PGJ2 (5 μM) served as positive controls for HMOX1 induction ability. *p < 0.05, **p < 0.01, ***p < 0.001, ****p < 0.0001 vs. control cells CON, control cells; SIM, simvastatin; CER, cerivastatin; FLU, fluvastatin; LOV, lovastatin; PRA, pravastatin; PGJ2, 15-deoxy-Δ-12,14-prostaglandin J2

To find out whether statins regulate HMOX activity in selected pancreatic cell lines in vitro, and whether this mechanism could possibly contribute to their anti-proliferative properties, we treated pancreatic cancer cells with individual statins for 12 h. However, none of the statins affected HMOX activity in any of the studied cell lines (Fig. 2a). There was no difference in basal HMOX activities between PA-TU-8902, MiaPaCa-2 and BxPC-3 cells (0.79 ± 0.12 vs. 0.89 ± 0.19 vs. 0.90 ± 0.14 nmol CO/h/mg protein, respectively, *p* > 0.05). For detailed analysis, we selected the most resistant cell line, PA-TU-8902, and tested the effect of cerivastatin, the most efficient statin (Fig. 1). Hemin and PGJ2, used as positive controls, strongly increased both expression and activity of HMOX after 4 and 12 h, respectively. Cerivastatin affected neither *HMOX1* expression nor HMOX activity in PA-TU-8902 cells. Addition of cerivastatin to hemin had no impact on HMOX induction by hemin itself (Fig. 2b, c).

### Hemin augments anti-proliferative effects of selected statins in pancreatic cancer cells

**Fig. 3:**
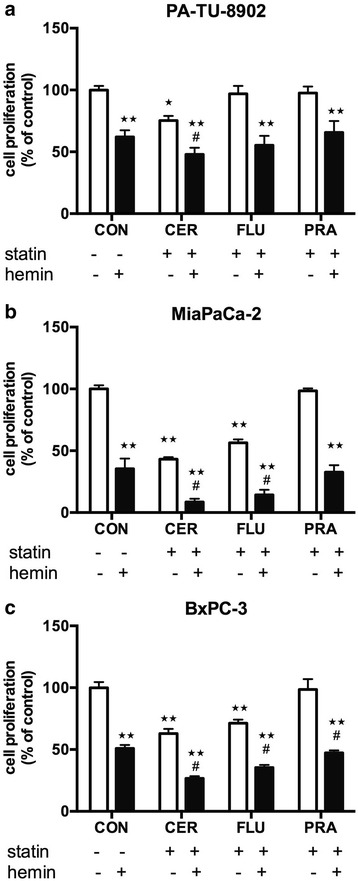
Effects of hemin and statins on proliferation of pancreatic cancer cells. The effects of hemin (30 μM), cerivastatin (12 μM), fluvastatin (12 μM) and pravastatin (12 μM) and their combination with hemin were measured in **a**) PA-TU-8902, **b**) MiaPaCa-2, and **c**) BxPC-3 after 48 h of treatment using CellTiter-Glo test. *p < 0.01, **p < 0.0001 vs. control cells, #*p* < 0.01 vs. hemin CON, control cells; CER, cerivastatin; FLU, fluvastatin; PRA, pravastatin

While using hemin primarily as a HMOX inducer, we unexpectedly noticed the decrease in proliferation in hemin-treated cells. Thus we further focused on more detailed analysis of possible anti-proliferative effects of hemin on the growth of pancreatic cancer cell lines. Indeed, we found a significant effect of 30 μM hemin on cell proliferation in all used cell lines after 48 h (Fig. 3), which was dose-dependent (data not shown). Hemin treatment decreased cell proliferation to 62 ± 5 %, 51 ± 3 %, and 38 ± 8 % in PA-TU-8902, BxPC-3 and MiaPaCa-2 cancer cells, respectively, with *p* < 0.0001 for all comparisons. Furthermore, we observed enhancement of anti-proliferative effects of statins by hemin, documented as decreased cell proliferation after 48 h of co-treatment (Fig. 3).

### Suppression of *HMOX* gene expression does not diminish anti-proliferative effects of cerivastatin and hemin

**Fig. 4:**
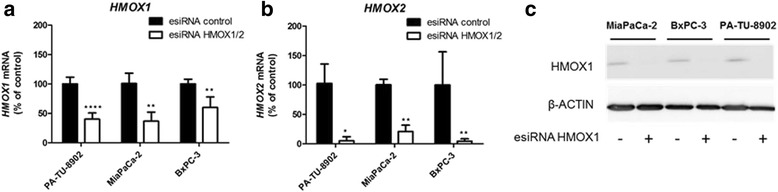
Effect of esiRNA *HMOX1/2* transfection on HMOX expression in pancreatic cancer cells. PA-TU-8902, MiaPaCa-2 and BxPC-3 cells were transfected with esiRNA for *HMOX1/HMOX2* or universal control esiRNA and mRNA expression of **a**) *HMOX1* and **b**) *HMOX2* was measured after 24 h. **c** HMOX1 protein expression was measured in cells treated with 30 μM hemin for 20 h. Hemin treatment was used to upregulate HMOX1 protein expression to cumulate detectable levels of HMOX1 protein. *p < 0.05, **p < 0.01, ****p < 0.0001 vs. esiRNA control cells

**Fig. 5:**
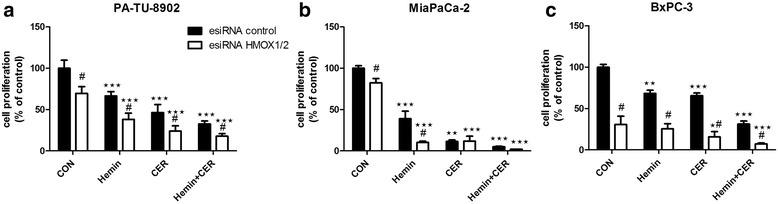
Effect of statin and hemin on proliferation of HMOX-silenced pancreatic cancer cells. Both *HMOX1* and *HMOX2* were silenced with esiRNA in **a**) PA-TU-8902, **b**) Mia-PaCa-2, and **c**) BXpC-3 pancreatic cancer cells, and the effect of cerivastatin (12 μM) and hemin (30 μM) on cell proliferation was measured by CellTiter-Glo test after 48 h of treatment. *p < 0.05, **p < 0.01, ***p < 0.0001 vs. control cells, #*p* < 0.01 vs. esiRNA control cells. CON, control cells; CER, cerivastatin

Recently it has been suggested, that statin treatment promotes post-transcriptional regulation of the HMOX1 protein rather than affecting *HMOX1* gene expression in human endothelial cells [30]. To further resolve if HMOX is involved in anti-proliferative effects of statins on human pancreatic cancer, we silenced *HMOX1* and *HMOX2* in pancreatic cancer cells with esiRNA and assessed cell proliferation after 48 h of exposure to cerivastatin and hemin. In all cancer cell lines, *HMOX1* and *HMOX2* were successfully silenced as demonstrated in Fig. 4. Significant anti-proliferative effect of cerivastatin was observed after 48 h in both silenced and cells transfected with esiRNA universal control. Interestingly, cell growth was affected by the silencing itself implying the important role of HMOX in the cell survival. Furthermore, hemin treatment effectively decreased cell proliferation in controls, as well as *HMOX*-silenced cells, and addition of hemin to cerivastatin endorsed the anti-proliferative effect of cerivastatin (Fig. 5), indicating that these processes are not mediated by HMOX.

### *Hemin and cerivastatin enhance ROS production and cell death* in vitro

**Fig. 6:**
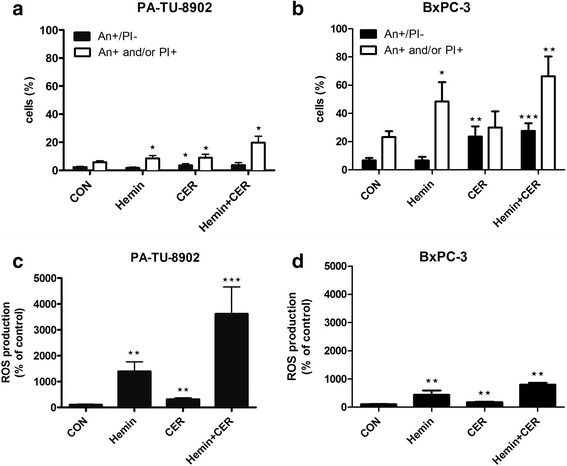
Effect of hemin and cerivastatin on ROS production and apoptosis in PA-TU-8902 and BxPC-3 pancreatic cancer cells. The effects of hemin (30 μM), cerivastatin (12 μM) and their combination on apoptosis in **a**) PA-TU-8902 and **b**) BxPC-3, and ROS production in **c**) PA-TU-8902 and **d**) BxPC-3 were measured after 48 h of treatment. An+/PI- represents cells in early apoptosis, An + and/or PI+ represents cells in late apoptosis or already dead. *p < 0.05, **p < 0.01, ***p < 0.0001 vs. control cells. CON, control cells; CER, cerivastatin

To evaluate the possible mechanism of cell growth suppression, we assessed apoptotic effects of hemin and cerivastatin in PA-TU-8902 and BxPC-3 cells. While there was no notable effect of hemin on early apoptosis, we found a significant increase in the total cell death rate including late apoptotic and post-apoptotic necrotic cells at 48 h of treatment. In fact, the major increase in cell death was noticed within the first 14 h of treatment with no significant elevation in the later phase (data not shown). Cerivastatin treatment caused a significant increase in all phases of apoptosis including the early phase (Fig. 6a, b). Since apoptotic pathways may be linked to ROS production, we assessed total ROS production after 48 h of treatment, and observed a substantial increase in ROS concentration in response to cerivastatin (314 ± 53 % and 173 ± 22 % for PA-TU-8902 and BxPC-3, respectively, *p* < 0.05 for both comparisons) and even much higher increase due to hemin (1394 ± 372 % and 441 ± 152 % for PA-TU-8902 and BxPC-3, respectively, *p* < 0.01 for both comparisons), with a further additive effect of both compounds (3615 ± 1043 % and 795 ± 72 % for PA-TU-8902 and BxPC-3, respectively, *p* < 0.01 for both comparisons) (Fig. 6b, c, d). Interestingly, the rate of cell death and ROS production differed between both cell lines suggesting cell death was not in direct relationship with ROS production.

### Cerivastatin prevents K-Ras protein trafficking in PA-TU-8902

**Fig. 7:**
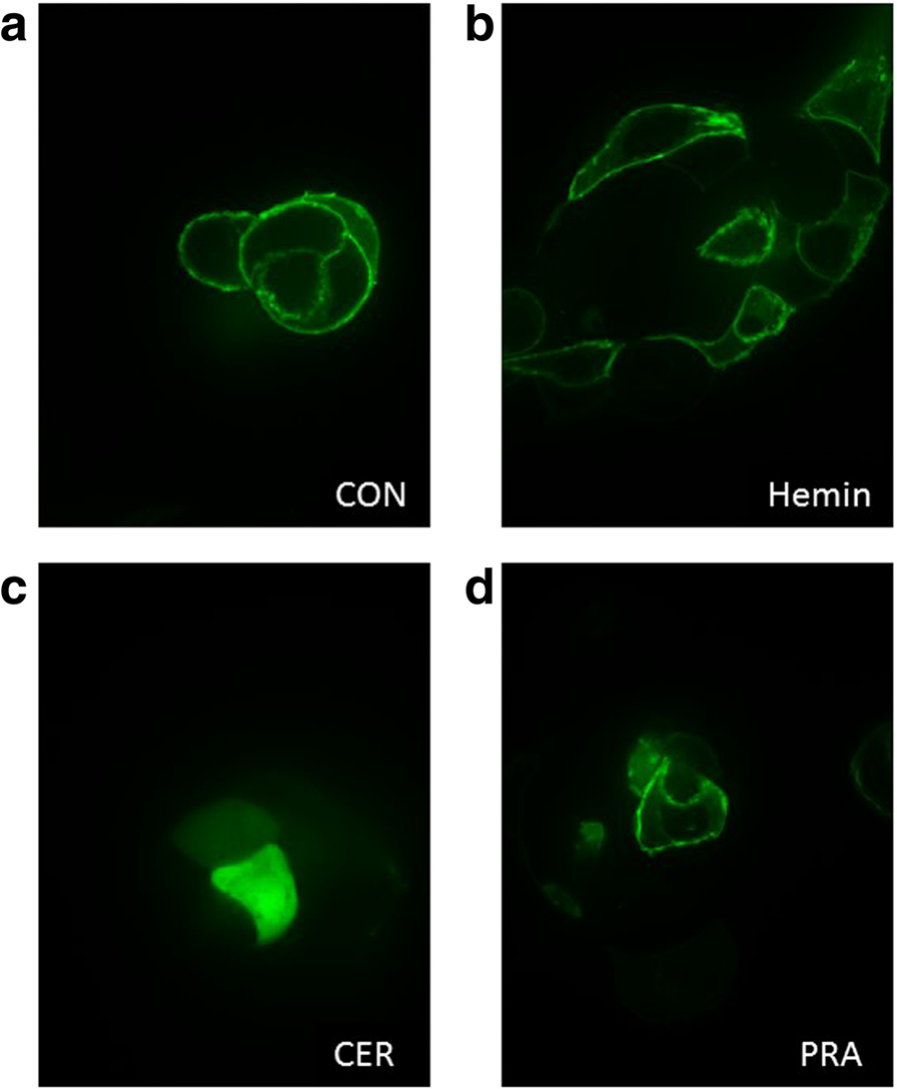
Effect of hemin and statins on GFP-K-Ras localization in PA-TU-8902 pancreatic cancer cells. The effect of **a**) vehicle, **b**) hemin (30 μM), **c**) cerivastatin (12 μM) and **d**) pravastatin (12 μM) on localization of GFP-K-Ras was tested in PA-TU-8902 pancreatic cancer cells transfected with pEGFP-K-RasWT plasmids after 24 h of treatment. CON, control cells; CER, cerivastatin; PRA, pravastatin

Since we previously reported that pravastatin is the only statin not being able to prevent GFP-K-Ras protein from accumulation on the cell membrane in MiaPaCa-2 cells [4], we decided to detect the effect of cerivastatin and pravastatin on localization of GFP-K-Ras protein also in the thoroughly tested PA-TU-8902 cell line. As demonstrated in Fig. 7, cerivastatin efficiently inhibited GFP-K-Ras protein trafficking from cytoplasm to the cell membrane, while pravastatin was inefficient. Hemin itself, or in combination with statins did not influence GFP-K-Ras trafficking at all (data not shown).

### Cerivastatin downregulates selected markers of invasiveness

**Fig. 8:**
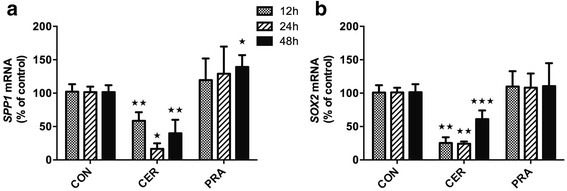
Effect of cerivastatin and pravastatin on selected markers of invasiveness in PA-TU-8902 pancreatic cancer cells. **a**) Osteopontin (*SPP1*) and **b**) *SOX2* mRNA expressions were measured in different time points of treatment with cerivastatin (12 μM) and pravastatin (12 μM). *p < 0.05, **p < 0.01, ***p < 0.001 vs. control cells. CON, control cells; CER, cerivastatin; PRA, pravastatin

Among others, poor prognosis of pancreatic cancer is due to early metastases and high invasiveness of this type of tumor. To determine whether cerivastatin treatment affects invasiveness of pancreatic cancer cells, we assessed selected markers previously reported to be associated with metastatic processes in pancreatic cancer, i.e. SPP1 and SOX2. In PA-TU-8902 cells, *SPP1* mRNA expression was significantly suppressed to 59 ± 13 % (*p* < 0.01) at 12 h of treatment with persisting effect of up to 48 h (17 ± 8 % and 40 ± 20 % at 24 and 48 h, respectively; *p* < 0.05; Fig. 8a). Similarly, *SOX2* mRNA expression decreased to 25 ± 9 % (*p* < 0.01) in 12 h with persisting effect to 48 h of treatment (24 ± 4 % and 61 ± 13 % at 24 and 48 h; respectively; *p* < 0.01, Fig. 8b).

## Discussion

Only little progress has been achieved in the treatment of pancreatic cancer over recent decades [31]. Among multiple tested experimental drugs, statins were shown to display anticancer effects in this malignant disease [32, 33]. In our study, we investigated a possible relation between statins and the HMOX pathway that play a role in pancreatic carcinogenesis [25].

Even though HMOX upregulation is associated with beneficial effects for cells, its role in carcinogenesis remains controversial [19]. HMOX seems to negatively affect the outcome of treatment [25] and enhance the aggressiveness and progression of pancreatic cancer [34]. Moreover, pancreatic cancer cells were shown to overexpress HMOX1 compared to normal pancreatic tissue [25, 34]. On the other hand, statins have been previously shown to upregulate *HMOX* gene expression, and some of their protective effects are believed to be mediated *via* this pathway [24]. Together with these HMOX1-inducing effects, statins simultaneously inhibit pancreatic cancer cell proliferation [4]. In this study, we assessed the overall relationship of particular statins to HMOX regulation in pancreatic cancer cells. We were able to demonstrate no effect of the tested statins on HMOX activity in selected human pancreatic cancer cell lines, despite their remarkable anti-proliferative effects. Moreover, suppression of proliferation by cerivastatin treatment persisted also in *HMOX1-* and *HMOX2-*silenced cells, indicating that these effects did not depend on the HMOX pathway. Importantly, we noted that *HMOX* silencing decreased the cell growth, implying an important role of HMOX in pancreatic cancer cell survival. This is in agreement with previous studies [25, 34].

To test a possible effect of HMOX on pancreatic carcinogenesis, hemin, a potent HMOX1 inducer [26], was used in further experiments. To our surprise, a significant decrease in cell proliferation was observed in all tested pancreatic cancer cell lines, despite HMOX induction. Moreover, co-treatment of the cells with hemin and statins increased the anti-proliferative effect of the latter. This is most likely due to hemin-mediated HMOX-independent mechanisms that play a role in cell proliferation and survival. Indeed, we found significant hemin-induced increase in apoptosis, corresponding to considerable increase in ROS production. The same cell growth inhibitory effects were observed even in *HMOX*-silenced cells, further suggesting the independence of hemin bioactivity on the HMOX pathway*.* Thus, neither statins- nor hemin-dependent suppression of proliferation involved the HMOX system; further, induction of HMOX by hemin did not prevent this response of pancreatic cancer cells to the agents. Nevertheless, our data from HMOX1/2 silencing support pro-carcinogenic role of HMOX in pancreatic cancer, consistent with previous clinical observation [25].

Despite the fact that hemin was suggested to contribute to increased colon cancer incidence in red meat eaters [35], other studies shown clear anticancer effects of this compound [36] supporting our findings.

Similarly as in our previous study [4] and as discussed recently [37], we observed remarkable differences in anti-proliferative effects of individual statins, which were dependent also on the cell line used. Both MiaPaCa-2 and PA-TU-8902 cells carry the *K*-*ras* mutation in codon 12 [28, 29]. Hamidi and colleagues found that in pancreatic cancer cells, this mutation makes the cells more sensitive to inhibitors of MEK1/2, which is a kinase activated by K-Ras [29]. To extend our study to pancreatic cancer cells lacking the *K*-*ras* mutation, we also used BxPC-3 cells carrying a wild-type *K*-*ras* proto-oncogene and overexpressing cyclooxygenase [28]. As expected, MiaPaCa-2 cells were the most susceptible and PA-TU-8902 were the most resistant cells to statin treatment in our experiments.

All the statins except pravastatin exerted remarkable growth inhibitory activity in all tested pancreatic cancer cell lines with cerivastatin being the most efficient of these agents. One of the mechanisms possibly involved in anti-carcinogenic effect of cerivastatin might be increased production of ROS, similarly as demonstrated in lymphoma cells [38]. Interestingly, this phenomenon was much more pronounced in hemin-treated cells and, in particular, in cells exposed to hemin together with cerivastatin.

The K-ras pathway is another possible target of statins. In fact, Gbelcova and colleagues demonstrated that statins, except for pravastatin, prevented the GFP-K-Ras protein from its cell membrane localization in MiaPaCa-2 cells [4]. We performed a similar experiment in PA-TU-8902 pancreatic cancer cells, and found that while pravastatin did not affect translocation of the K-Ras protein to the cell membrane, cerivastatin significantly prevented GFP-K-Ras from membrane localization. The K-Ras signaling pathway is essential for metastatic lesion formation and tumor invasiveness [39]. Interestingly, cerivastatin but not pravastatin treatment of PA-TU-8902 cells significantly decreased the expression of *SPP1* and *SOX2*, factors with important role in cancer metastasis and aggressiveness [40, 41]. Meta-analysis of 11 studies revealed that patients with pancreatic cancer have elevated serum levels of SPP1 [42]. Similarly, *SOX2* over-expression promotes self-renewal and de-differentiation of pancreatic cancer cells [43]. In our experiments, both markers were downregulated in pancreatic cancer cells exposed to cerivastatin, pointing to a lowering effect of the statin on the metastatic potential of the cells.

## Conclusion

Our data suggest that anti-proliferative effects of statins are not mediated *via* HMOX pathway. Cerivastatin, the most efficient statin in our study, was capable of inducing several ‘events’ involved in carcinogenesis, including apoptosis, ROS production and inhibition of K-Ras trafficking. Hemin treatment not only substantially decreased cell proliferation independently on HMOX induction, but enhanced anti-proliferative properties of statins in human pancreatic cancer cells. Our findings support the role of statins as agents with potential anti-pancreatic cancer activities.

### Ethics approval and consent to participate

Not applicable

### Consent for publication

Not applicable

### Availability of data and materials

The datasets supporting conclusions of this article are included within the article.

## Abbreviations

DMEM: dulbecco’s Modified Essential Media
FBS: fetal bovine serum
GFP: green fluorescent protein
HMG CoA: 3-hydroxyl-methylglutaryl coenzyme A
HMOX: heme oxygenase
NADPH: reduced nicotinamide adenine dinucleotide
PGJ2: 15-deoxy-Δ-12,14-prostaglandin J2
SPP1: secreted phosphoprotein 1, osteopontin
SOX2: sex-determining region Y-related HMG box 2
